# Treatment of asthma in young children: evidence-based recommendations

**DOI:** 10.1186/s40733-016-0020-z

**Published:** 2016-03-02

**Authors:** Jose A. Castro-Rodriguez, Adnan Custovic, Francine M. Ducharme

**Affiliations:** 1 Division of Pediatrics, School of Medicine, Pontificia Universidad Católica de Chile, Lira 44, 1er. Piso, casilla 114-D, Santiago, Chile; 2 Imperial College London, Department of Paediatrics, St Mary’s Campus Medical School, Room 244, Norfolk Place, London, W2 1PG England; 3 Department of Paediatrics, University of Montreal, Montreal, Canada; 4 Research Centre, CHU Sainte- Justine, Montreal, Canada

**Keywords:** Wheezing, Asthma, Treatment, Predictive index, Infants, Preschoolers

## Abstract

In the present review, we focus on evidence-based data for the use of inhaled corticosteroids (ICS), leukotriene receptor antagonist (LTRA), long-acting beta2-agonits (LABA) and oral corticosteroids (OCS), with a special emphasis on well-performed randomized clinical trials (RCTs) and meta-analyses of such trials for the chronic management of asthma/wheeze in infants and preschoolers. Results: Seven meta-analyses and 14 RCTs were reviewed. Daily ICS should be the preferred drug for infants/preschoolers with recurrent wheezing, especially in asthmatics. For those with moderate or severe episodes of EVW, the use of high intermittent ICS doses significantly reduce the use of OCS. There is no evidence of effect of intermittent ICS at low-moderate dose in preschoolers with mild EVW episodes. In preschoolers with asthma, there were no significant differences between daily vs. intermittent ICS in terms of asthma exacerbations with insufficient power to conclude to equivalence; however, for other asthma control outcomes, daily ICS works significantly better than intermittent ICS for older children. Daily ICS is superior to daily or intermittent LRTA for reducing symptoms, preventing exacerbations, and improving lung function. No RCTs testing combination therapy with ICS and LABA (or LTRA) were published in infant/preschoolers. Parent-initiation of OCS at the first sign of symptoms is not effective in children with recurrent wheezing episode. In terms of ICS safety, growth suppression is dose and molecule-dependent but it’s effect is not cumulative beyond the first year of therapy and may be associated with some catch-up growth while on or off therapy. Linear growth must be monitored as individual susceptibility to ICS drugs may vary considerably.

## Background

Wheezing is a common symptom in the first years of life, but a minority of children will continue to experience wheezing symptoms in school years and beyond [1]. Based on the epidemiologic data on the natural history and temporal patterns of wheezing, several childhood wheezing phenotypes have been described, with different risk factors somewhat associated with each phenotype. However, the use of these “epidemiologic” phenotypes such as transient early, prolonged early, persistent (atopic and non-atopic), late-onset, and intermediate-onset wheezing, is limited, since they can only be identified retrospectively; indeed, they were defined using statistical inference on longitudinally collected data, and not useful in the present as they are defined by events that will occur in the future [2]. Thus, it has been proposed that wheezing phenotypes be based on the trigger(s) and temporality of symptoms (such as episodic viral wheeze [EVW] and multiple-trigger wheeze [MTW]) which can be ascertained in a clinic and could be more practical to make treatment decisions [3]. However, classification of preschool wheezers into EVW or MTW may change in up to 50 % of cases within a 1-year period, suggesting that these phenotypes overlap considerably, perhaps as children show and parents observed, more symptoms with time or conversely show improvement with therapy [4].

Effective management options for early-life asthma/wheezing would be of great importance for a number of reasons. First, the burden of disease is greatest in preschoolers with a significantly higher proportion of emergency department (ED) visits, more hospitalizations, more sleep disturbances and more limitation of family activities/play, than older children [5, 6]. Secondly, the irreversible impairment in lung function may occur during the preschool period, suggesting a window of opportunity to perhaps prevent irreversible damage; [7] It is possible that the repeated and cumulative lung injury caused by various respiratory infections (e.g., rhinovirus, respiratory syncytial virus, etc.) that are frequent at this age maybe causal or important intercurrent factors affecting lung growth and asthma persistence. Perhaps, one of the reasons of that high morbidity among preschoolers is that the diagnosis of asthma without lung function testing is challenging, resulting in wide variation in treatment approaches, compounded by the paucity of evidence.

In the past 15 years, the diagnosis of asthma has hinged on the ability to predict persistence of asthma at 6 years. Several asthma predictive rules have emerged. The Asthma Predictive Index (API) [8], originally developed in the Tucson cohort study, is the most widely used. The API is simple and cheap, and its major strength is its good positive likelihood ratio ~7.4 (the post-test probability of disease can improved from 2 to 7 times) and high specificity (~97 %) [9].

In the present review, we will focus on chronic management of asthma/wheeze in preschoolers. We review the evidence (RCTs and systematic reviews with meta-analyses) for the use of inhaled corticosteroids (ICS), leukotriene receptor antagonist (LTRA), oral corticosteroids (OCS), and long-active beta-2 agonists (LABA). We present the most relevant data of the meta-analysis performed for each specific topic and described the RCTs not included in those meta-analyses. When no meta-analysis was performed, RCTs were described. In this review, we specifically did not consider the acute management of asthma/wheeze at home, during emergency department (ED) visits or hospitalizations. We acknowledge that these trials (RCTs) generally include heterogeneous group of participants, with differences in age, inclusion criteria, triggers, severity, and possibly diagnoses.

## Results

### Data extraction

We (JACR) performed a searched in PubMed and the Cochrane Library with the keywords: (asthm* or wheez*) AND (inhaled corticosteroids or corticosteroids or leucotriene or leukotriene or montelukast or long-acting beta agonists), limited for clinical trials or systematic reviews and for infants or preschool children. We included 7 systematic reviews with meta-analysis of RCTs and 14 RCTs not included in the systematic reviews.

#### Inhaled corticosteroids

##### Daily ICS

Wilson et al. [10] performed a parallel study randomized 161 patients with EVW to ICS (budesonide 400 μg/day) or placebo administered over the course of four months, and could not demonstrate any significant of the active treatment on the use of rescue OCS, admission to hospital, overall symptom scores, number of symptom-free days, severity of symptoms, or duration of episodes between treatments when they compared vs. placebo.

**Fig. 1:**
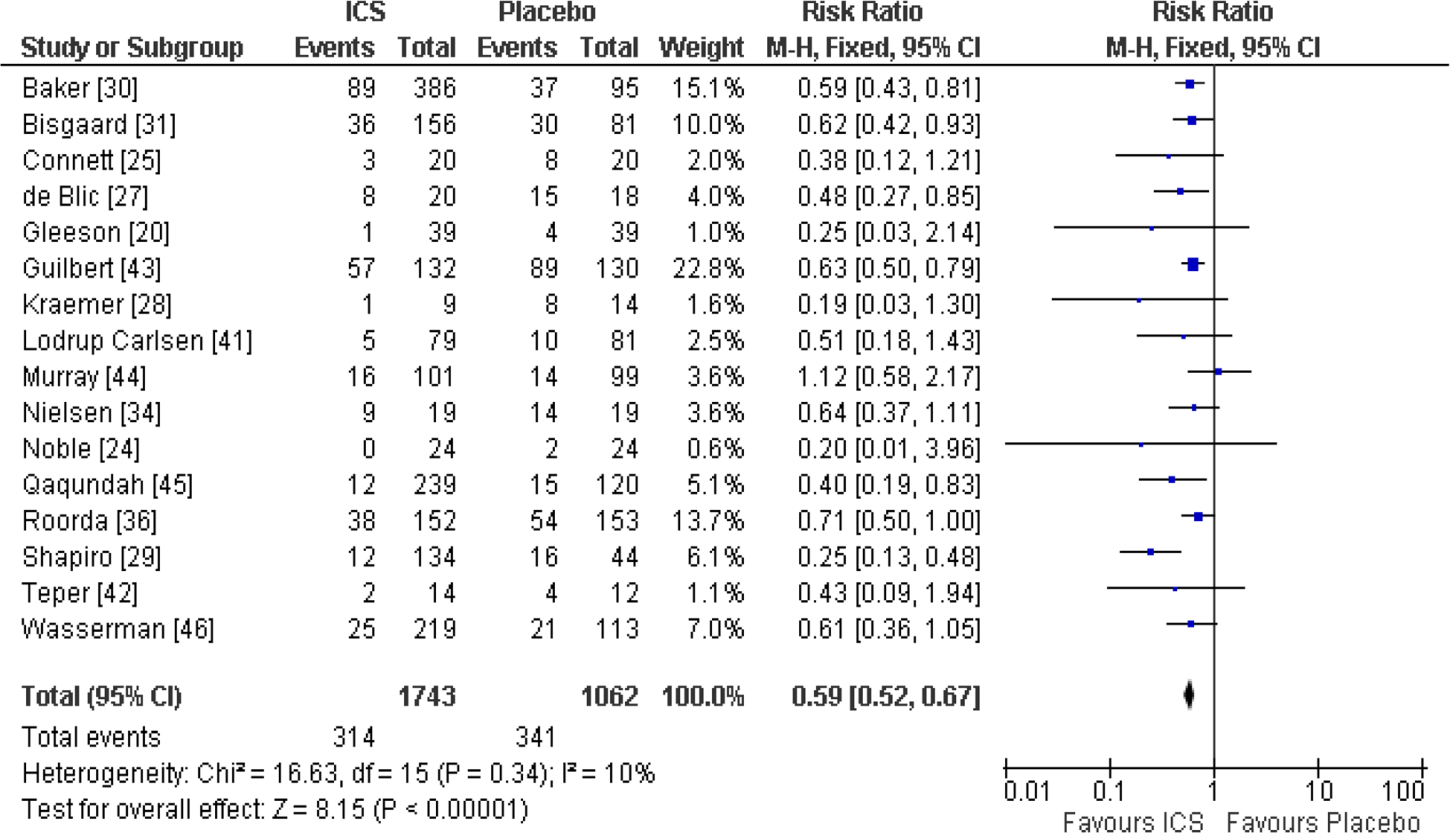
Pooled RRs (with 95 % CI) for wheezing/asthma exacerbations of eligible studies comparing ICSs vs. placebo in infants or preschoolers [11]. (reproducing with the author’s permission)

High quality evidence supports the use of ICS in infants or pre-school children with recurrent wheezing or asthma for at least 6 months before study entry. Castro-Rodriguez and Rodrigo [11] conducted a meta-analysis on 29 RCTs (*n* = 3592) to compare the efficacy of ICS vs. placebo in infants and preschoolers with recurrent wheezing or asthma. They reported that patients who received ICS had significantly less wheezing/asthma exacerbations requiring OCS than those on a placebo (RR = 0.59, 95 % CI [0.52–0.67], *p* = 0.0001, I^2^ = 10 %), and with a NNT = 7 [6–9], (Fig. 1). Post-hoc subgroup analysis suggests that although ICS is effective in asthma and recurrent wheezing, but with stronger effect in those with a diagnosis of asthma than wheezing (interactive test RR = 0.76 [0.58–0.99], *p* = 0.04); but was independent of age (infants vs. preschoolers), atopic condition, type of inhaled corticosteroid (budesonide vs. fluticasone), mode of delivery (metered-dose inhaler [MDI] vs. nebulizer), and study quality and duration (<12 vs. ≥ 12 weeks). In addition, children treated with ICS had significantly fewer withdrawals caused by wheezing/asthma exacerbations, less albuterol use, and more clinical and functional improvement than those on placebo.

In conclusion daily treatment with ICS is consistently appears as an effective strategy in preschoolers with recurrent wheezing, especially those with asthma diagnosis, including those with EVW.

##### Intermittent ICS

**Fig. 2:**
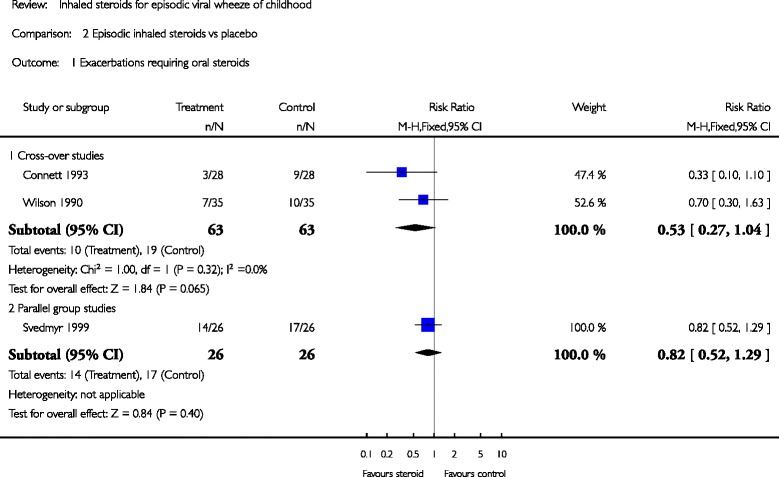
Pooled RRs (with 95 % CI) for exacerbation requiring oral steroids of eligible studies comparing episodic ICS vs placebo in infants or preschoolers [12]. (reproducing with the author’s permission)

A Cochrane review conducted by McKean & Ducharme [12] with 3 RCT (*n* = 122 preschoolers with EVW), 2 cross-over and 1 parallel study comparing high ICS doses (1.6–2.25 mg per day) show a reduction in requirement for OCS among those with ICS vs. placebo (RR = 0.53 [0.27–1.04] for the 2 cross-over studies, and RR = 0.82 [0.52–1.29] for the parallel study), (Fig. 2). In terms of ED/doctor visits, only one study reported this outcome and shows an effect favoring ICS (RR = −0.70 [0.50 to 0.97]); in terms of hospital admissions, the effect of ICS was not significant different compared with placebo. However, a recently published parallel RCT [13] comparing intermittent beclomethasone by nebulizer (400 μg twice daily) vs. placebo for 10 days in 525 Italian preschoolers with mild EVW (had at least 1 episode of viral wheezing diagnosed by a physician in the preceding 12 months, and had no or minimal asthma-like symptoms between distinct airway infections) showed no group difference in % of wheezing diagnosed by the pediatrician during a upper respiratory tract infection episode (primary outcome) nor reducing severity of wheezing, asthma-like symptoms score, extra visits, ED attendance, prescription of rescue drugs.

**Fig. 3:**
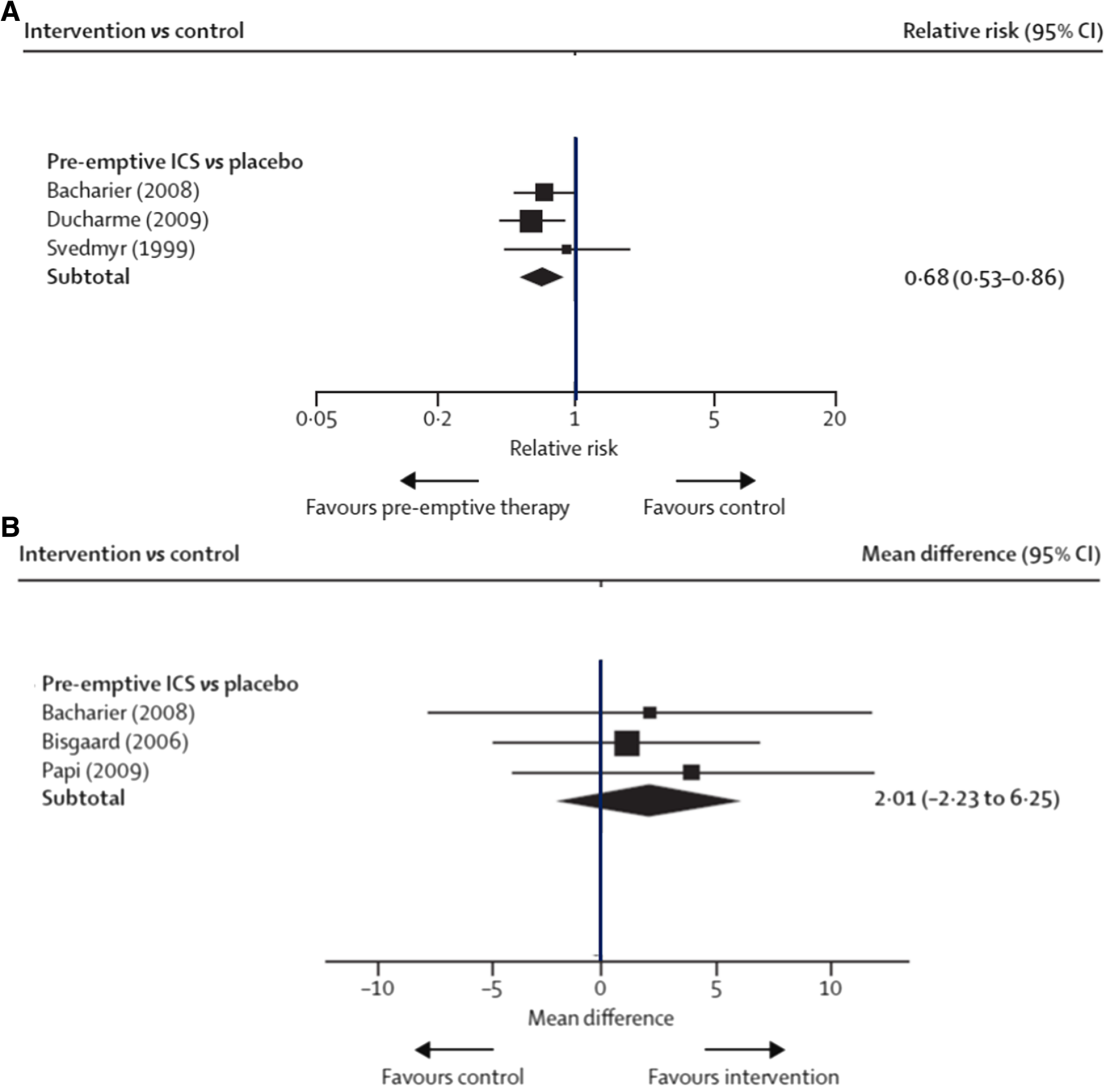
**a** Pooled RR (with 95 % CI) for exacerbations needing rescue oral steroids of eligible studies comparing pre-emptive ICS vs. placebo in infants or preschoolers [14]. (reproducing with the author’s permission). **b** Mean group difference (with 95 % CI) of percentage of asthma-free days of eligible studies comparing pre-emptive ICS vs. placebo in infants or preschoolers [14]. (reproducing with the author’s permission)

Recently, Ducharme et al. [14] performed a meta-analysis of 4 new RCTs (*n* = 1024) reporting a reduced risk of exacerbations need rescue OCS in preschoolers with moderate or severe EVW using high ICS doses vs. placebo (RR = 0.68 [0.53–0.86], but the difference did not reach significance in asthma-free days (MD = 2.01 [−2.23–6.25], (Fig. 3a and b).

In conclusion, for infants and preschoolers with EVW and recurrent wheezing, the use of high intermittent ICS doses significantly reduce the use of OCS in those with moderate or severe episodes; no apparent effect of intermittent low-moderate doses of ICS in children with mild EVW episodes.

##### Daily vs. intermittent ICS

A meta-analysis conducted by Rodrigo & Castro-Rodriguez [15] evaluated daily vs. intermittent ICS among preschoolers (2 RCTs, *n* = 498), school-age children (2 RCTs, *n* = 259) and adults (3 RCTs) with persistent wheezing and mild to moderate stable persistent asthma. There was no statistically significant difference in the rate of asthma exacerbations between the two strategies in all patients (RR = 0.96 [0.86–1.06], *p* = 0.40, I^2^ = 0 %). Sub-group analysis did not identify significant group differences regarding age, duration of studies, or step-up strategy. However, the daily ICS group (in all patients) had a significant greater increase in asthma-free days (RR = 1.10 %, 95 % CI: [1.01 to 1.20], *p* = 0.03, I^2^ = 10 %, NNT = 22, 95 % CI [9 to58]) than those treated with intermittent high-dose ICS. There were no significant differences in rescue medication use, exhaled nitric oxide measurement, and children’s linear growth rate between daily and intermittent ICS.

**Fig. 4:**
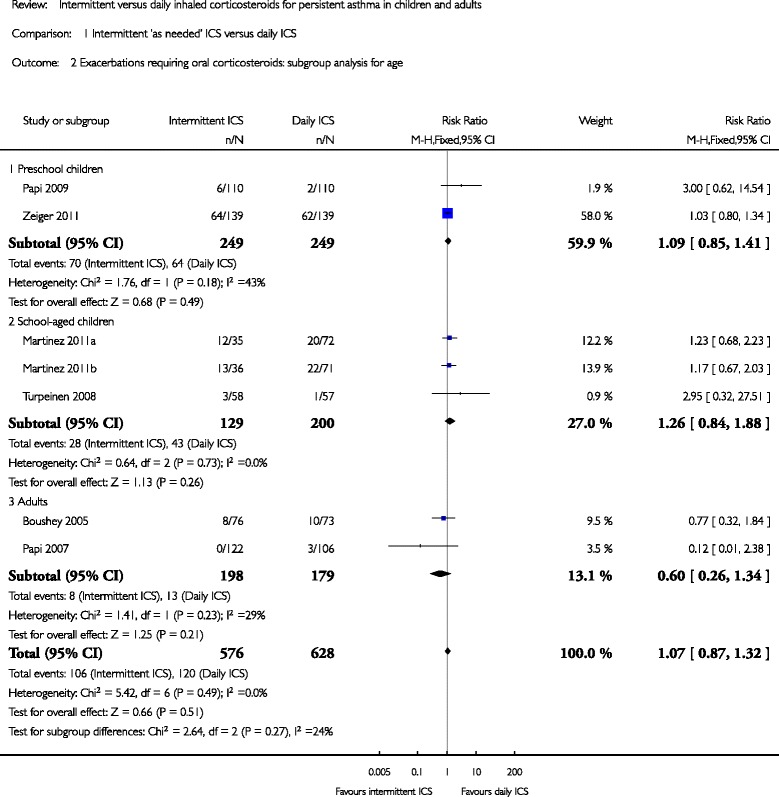
Pooled RR (with 95 % CI) for exacerbation requiring oral corticosteroids of eligible studies comparing intermittent ICS vs. daily ICS in infants or preschoolers [16]. (reproducing with the author’s permission)

Later, a Cochrane meta-analysis by Chauhan et al. [16] compared the efficacy and safety of intermittent vs. daily ICS for the management of children and adults with persistent asthma published October 2012. The review included 2 RCTs involving 498 preschoolers (the same studies included by the former meta-analysis) and 3 involving school-age children (5–18 years) and 2 adult trials. When analyzing patients of all ages, the daily ICS group was associated with a statistically significant improvement in the change from baseline PEFR, more symptom-free days, more asthma control days, less use of rescue medication, and a greater reduction in the change from baseline in exhaled nitric oxide vs. intermittent ICS group. In the subgroup analysis focusing on preschoolers, there were no significant difference in exacerbations requiring OCS among intermittent vs. daily ICS (RR = 1.26 [0.84–1.88], *p* = 0.49, I^2^ = 43 %), but there is insufficient evidence to conclude to equivalence. (Fig. 4). Among preschool and school-aged children, intermittent ICS were associated with greater growth by 0.41 cm change from baseline (*p* = 0.004) compared to daily treatment.

In conclusion, there were no significant differences between daily vs. intermittent ICS in terms of asthma exacerbations but insufficient data to conclude to equivalence. however, for other asthma control outcomes, daily ICS works significantly better than intermittent ICS for older children.

##### Safety of ICS

Of the adverse effects associated with ICS, growth suppression is the most concerning for physician and parents. Most RCT focused on pre-pubertal school-aged children. Only few RCTs on preschoolers reported this adverse effect. Specifically, in a RCT on daily low dose (fluticasone 100 μg twice daily) vs. sodium cromoglycate for 52 weeks, there was no significant difference in mean adjusted growth rates between the two groups: 84.0 mm/year vs. 86.4 mm/year, respectively (difference: −2.4 mm/year, 95 % CI −6.6 to 1.8) [17]. In a RCT on intermittent high-dose (fluticasone 750 μg twice daily for 10 days), the difference in height between ICS and placebo was also small −0.61 cm [−1.31 to 0.09] [18].

**Fig. 5:**
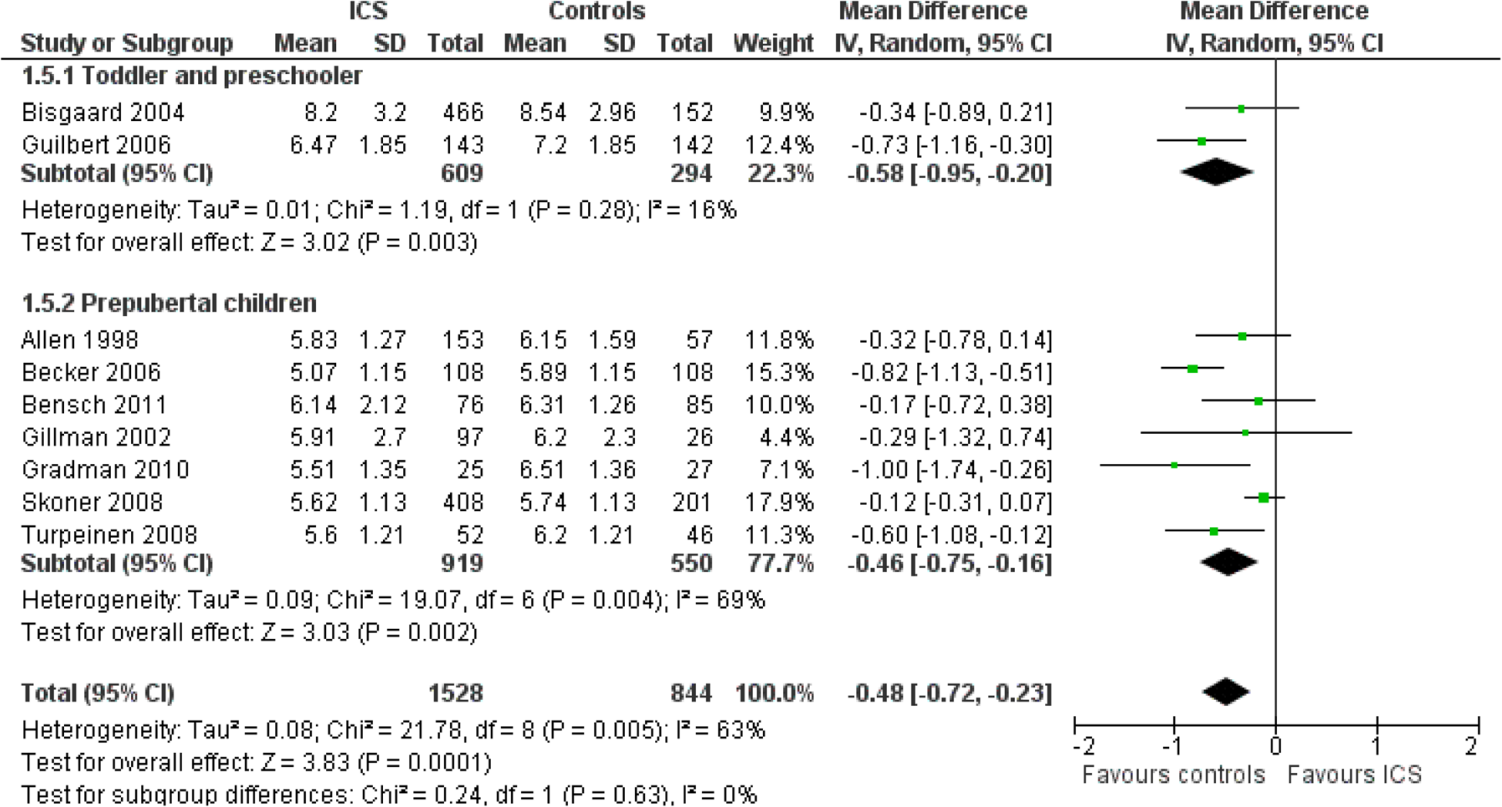
Mean difference (with 95 % CI) of change from baseline in height (cm) during one year of treatment comparing no steroids drugs vs. ICS [19]. (reproducing with the author’s permission)

In predominantly school-aged children with mild to moderate persistent asthma, a recent systematic review by Zhang et al. [19] showed that regular use of ICS at low or medium daily doses was associated with a statistically significant growth suppression measured by linear growth velocity, change from baseline in height, and change in height SDS during a one-year treatment period. Compared with placebo or non-steroidal drugs, ICS was associated with a statistically significant reduction in linear growth velocity (*N* = 14 RCTs, *n* = 5717 participants, MD −0.48 cm/yr [−0.65 to −0.30], moderate quality evidence) and in the change from baseline in height (*N* = 15 RCTs, *n* = 3275 participants, MD −0.61 cm/y [−0.83 to–0.38], moderate quality evidence) during a one-year treatment period. In the subgroup of toddlers or infants (*N* = 2, *n* = 903), the change in the baseline of height (cm) during one year of treatment was of similar magnitude than that of placebo: MD −0.58 [−0.55 to −0.20], *p* = .003, I^2^ = 16 %, (Fig. 5). The subgroup analysis on 14 RCTs indicated that the effect size of ICS on linear growth velocity appeared to be associated more strongly with the ICS molecule (with apparent greater suppression of beclomethasone, budesonide and probably mometasone compared to ciclosenide, fluticasone) than with the device or dose. ICS-induced growth suppression seemed to be maximal during the first year of therapy and less pronounced during subsequent years of treatment. Although catchup growth up to 12 months after ICS cessation has been documented, limited evidence suggests that ICS-induced growth suppression in pre-pubertal school-aged children with prolonged daily ICS therapy may persist until they reach adult height. Indeed, a trial with follow-up into adulthood showed that participants of pre-pubertal age treated with budesonide 400 μg/day for a mean duration of 4.3 years had a mean reduction of 1.20 cm [−1.90 to −0.50] in adult height compared with those treated with placebo [20].

In conclusion, ICS may be associated with growth suppression although the evidence is limited in preschoolers. In pre-pubertal school-aged children, the growth suppression appears neither progressive nor regressive, and it is not cumulative beyond the first year of therapy. Consequently, it is prudent to monitor linear growth in all children treated with ICS, irrespective of age, given that individual susceptibility to these drugs may vary considerably and select molecules with the less growth suppression and lower the dose to the minimal effective one.

#### LTRA (daily and intermittent)

**Fig. 6:**
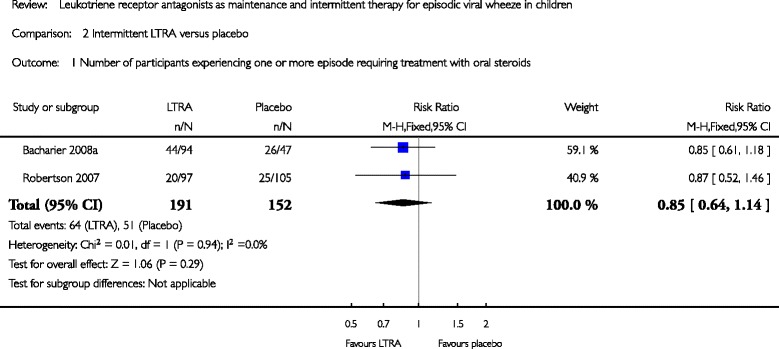
Pooled RR (with 95 % CI) for number of participants experiencing one or more episode requiring treatment with oral corticosteroids of eligible studies comparing intermittent LRTA vs. placebo in infants or preschoolers [21]. (reproducing with the author’s permission)

A recent Cochrane review [21] of RCTs with a parallel-group or cross-over (for intermittent LTRA only) design evaluated the evidence for the efficacy and safety of maintenance (more than 2 months) and intermittent (<14 days) LTRAs in the management of EVW in preschoolers. Five RCTs (one investigated maintenance treatment, three intermittent therapy and one had both maintenance and intermittent treatment arms) included 3741 participants. Each study involved oral montelukast and was of good methodological quality. For maintenance treatment, specific data obtained from a single study (*n* = 341), limiting to children with only an EVW phenotype, showed no statistically significant group reduction in the number of episodes requiring rescue oral corticosteroids associated with daily montelukast versus placebo (OR = 1.20 [0.70 to 2.06], moderate quality evidence). For intermittent LTRA, pooled data (*n* = 343) showed no statistically significant reduction in the number of episodes requiring rescue OCS in children treated with LTRA versus placebo (OR = 0.85 [0.64 to 1.14], *p* = 0.29, I^2^ = 0), (Fig. 6). Specific data for children with an EVW phenotype obtained from a single study (*n* = 963) of intermittent montelukast treatment showed a small, but statistically significant reduction in unscheduled medical attendances due to wheeze (RR = 0.83 [0.71 to 0.98]). For maintenance compared to intermittent LTRA treatment, no data relating to the primary outcome (one or more viral-induced episodes requiring treatment with OCS) of the review were identified. There were no other significant group differences identified in other secondary efficacy outcomes for maintenance or intermittent LTRA treatment versus placebo, or maintenance versus intermittent LTRA treatment. No differences on adverse events were found.

Knorr et al. [22] randomly assigned 689 children (aged 2–5 years) with a history of physician-diagnosed asthma to 12 weeks of treatment with montelukast or a placebo. Montelukast produced significant improvements compared with a placebo in daytime and overnight asthma symptoms, the percentage of days without asthma, the need for rescue bronchodilator or OCS use, physician global evaluations, and peripheral blood eosinophils.

Recently, a multicentre parallel-group randomized placebo-control trial [23], was carried out in 1358 children (aged 10 months to 5 years) with two or more wheeze episodes who were stratified by genotype into either a 5/5 or 5/x or x/x ALOX5 (arachidonate 5-lipoxygenasae) promoter genotype stratum, then randomly assigned to receive intermittent montelukast or placebo given by the parents at each wheeze episode over a 12 month period. There was no difference in unscheduled medical attendances for wheezing episodes (primary outcome) between children in the montelukast and placebo groups. Compared with placebo, unscheduled medical attendance for wheezing episodes were reduced in children given montelukast in the 5/5 stratum (IRR 0.80, 95 % CI 0.68–0.95, *p* = 0.01), but not in the 5/x + x/x stratum. The data suggest no clear benefit of intermittent montelukast in groups of young children with wheeze; however, the 5/5 ALOX5 promoter genotype might identify a montelukast-responsive subgroup.

In conclusion in preschoolers with EVW, there is no evidence of benefit associated with maintenance or intermittent LTRA treatment, compared to placebo, for reducing the number of children with one or more episodes requiring rescue oral corticosteroids, and little evidence of significant clinical benefit for other secondary outcomes. Therefore until further data are available, LTRA should be used with caution in individual children. It is likely that children with an apparent EVW phenotype are not a homogeneous group and that subgroups may respond to LTRA treatment depending on given genotype and the exact patho-physiological mechanism involved.

#### ICS vs. LTRA

At present, only few RCTs comparing daily ICS vs. LTRA in infants/preschoolers were performed. Kooi et al. [24] completed a small RCT (*n* = 63 Dutch children aged 2–6 years. with asthma-like symptoms) comparing daily fluticasone (100 μg twice daily) vs. montelukast (4 mg/day) vs. placebo for three months. Despite lack of power, the results suggest that fluticasone has a significant greater beneficial effect on symptoms than placebo, while montelukast significant decreased the blood eosinophil level compared to placebo. Children on fluticasone had significant better lung function parameters (lower airway resistance) than those on montelukast.

Krawiec et al. [25] carried out a small study in Poland in which 70 children aged 6 to 36 months with one to three wheezing episodes were randomized to receive either montelukast (4 mg), fluticasone (50 or 100 μg /day, <12 or ≥ than 12 months of age respectively) or no treatment for 12 weeks. There were no significant differences in primary outcome (number and percentage of wheezing episode within one year) between groups.

A RCT [26] on 2400 Pakistani children age 6 months to 5 years (mean age of 2.4 ± 1.25 years) with uncontrolled asthma randomized participants to ICS (200 μg /day) or montelukast (4 mg in children under one year of age, and 5 mg in those older) for 6 months. Failure of treatment was consider if patients were admitted to ED due to wheezing exacerbation. After 6 months of treatment, 51.6 % of children on ICS vs. 16.7 % on montelukast stepped-down therapy. On the other hand, 6.4 % of children on ICS vs. 32.1 % on montelukast were stepped-up therapy. Only one patient on ICS and two on montelukast were admitted to ED. However, no more details were found in the publication, limiting the interpretation.

Li YQ et al. [27] completed the first RCT comparing children with positive and negative API. In this trial, 239 wheezing Chinese children aged 17 to 60 months of age were divided into API-positive (*n* = 126) and API-negative groups (*n* = 113). Each group was randomly assigned to budesonide suspension or montelukast for four weeks. Asthma symptom scores were assessed and recorded at different time points. In the first four weeks of treatment, budesonide and montelukast were effective on symptoms among children with API positive and API negative. After 24 weeks of treatment, montelukast works better on symptoms than budesonide among children with API positive; however, both drugs works equally among children with API negative.

In conclusion, only few RCTs were performed comparing daily ICS vs. montelukast in infant/preschoolers, with no clear superiority between those drugs. However, at this moment, no RCT was published in a cross-over comparison of ICS vs LTRA in infants/preschoolers with recurrent wheezing and positive or negative API.

#### Intermittent OCS at home

Since children with EVW have episodic exacerbations triggered by viral respiratory infections, another therapeutic strategy for treating children with EVW consists of keeping the OCS at home, and having parents commence their use at the first sign of symptoms, without waiting for a medical review, in an effort to abort the attack. Short courses (3–5 days) of OCS (generally prednisolone) are commonly administered in this way.

In a crossover RCT study [28] 86 children (aged 2–4 years.) from a primary-care clinic and ED of an inner-city teaching hospital who had made two or more outpatient (ED or primary-care clinic) visits for acute asthma in the preceding year were enrolled for 12 months (6 months prednisone [2 mg/k up to 60 mg) or 6 months placebo). Parents were instructed to give their child one capsule for an asthma attack that had not improved after a dose of the child’s regular acute asthma medicine. Neither the total number of attacks nor the number for which medicine was used differed significantly by arm of study. There was a larger number of attacks resulting in outpatient visits when children were in the group that received prednisone (1.1 ± 0.59 versus 0.59 ± 0.86). This trend was less pronounced but persisted when limited to attacks for which the medicine was given (0.58 ± 0.99 versus 0.35 ± 0.55). Neither the number of attacks resulting in admission nor the number of hospital days differed significantly by arm of study. Oommen et al. [29] studied 217 children aged 1 to 5 years admitted to hospital with EVW who were randomized for parent-initiated prednisolone (20 mg once daily for 5 days) or a placebo for the next episode. The children were stratified according to amounts of serum eosinophil cationic protein and eosinophil protein X. As daytime and nighttime respiratory symptom scores and the need for hospital admission did not differ between treatment groups, and no effect of eosinophil priming was seen, the authors concluded that there is no clear benefit attributable to a short course of parent-initiated prednisolone for viral wheeze in children aged 1–5 years.

In conclusion it seems that therapeutic strategy for treating children with EVW consists of keeping the OCS at home and having parents commence their use at the first sign of symptoms is not effectiveness.

#### Long-acting beta2-agonists and LTRA as adjunct therapy to ICS

No RCT in this specific age group was published yet for adjunct therapy to ICS in preschoolers. Only a small retrospective study using fluticasone propionate (88–440 μg /day) + salmeterol in 50 preschoolers demonstrated a significant decrease in wheezing frequency and healthcare utilization (ED visits and hospitalization) compared to their previous treatment (ICS and/or LTRA) [30]. Therefore, RCTs comparing LABA or LTRA + ICS vs. ICS alone need to be performed before this combination therapy will be use in infant/preschoolers.

#### Asthma guidelines (Table 1)

**Table 1:** Summary of the stepwise approach for managing asthma in children less than 5 years of age according to different guidelines

	NAEPP [31]	British [32]	GINA [33]	Canadian [34]
Step 1	SABA prn	SABA prn	SABA prn	SABA prn
Step 2	Pref: Low-dose ICS	Pref: ICS 200–400 μg /day^a,b^	Pref: Daily low-dose ICS	Pref: Daily low-dose ICS
	Alter: cromolyn or LTRA	Alter: LTRA	Alter: LTRA or intermittent ICS	Alter: LTRA
Step 3	Medium-dose ICS	ICS + LTRA	Pref: Double low-dose ICS	Medium-dose ICS
			Alter: Low-dose ICS + LTRA	
Step 4	Medium-dose ICS + either LABA or LTRA	Refer to respiratory pediatrician	Pref: Continue controller & refer for specialist assessment	Referral to asthma specialist
			Alter: Add LTRA, increase ICS frequency, intermittent ICS.	
Step 5	High-dose ICS + either LABA or LTRA			
Step 6	High-dose ICS + either LABA or LTRA			
	Consider OCS			

*Alter* alternative, *GINA* Global Initiative for Asthma, *ICS* inhaled corticosteroids, *LABA* long active beta-2 agonists, *LTRA* leukotriene receptor antagonist, *NAEPP* National Asthma Education and Prevention Program, *OCS* oral corticosteroids, *Pref* preferred, *prn* pro re nata, *SABA* short active beta-2 agonist

^a^beclometasona dipropionate or equivalent doses

^b^Higher nominal doses may be required if drug delivery is difficult

The NAEPP [31], British [32], GINA [33] and Canadian [34] guidelines recommend that children under 5 years of age with mild intermittent symptoms (step 1) should be treated with short-acting beta 2-agonits (SABA) alone. Those with persistent disease (step 2) should be treated with controller therapy: all guidelines identify daily low-dose ICS as the preferred controller, with LRTA suggested as an alternative therapy [31–34]. But if the asthma is not controlled with low-dose ICS, the recommendation for the step 3 varies according to the guidelines (without any supporting trial): medium-dose ICS [31, 33], or low-dose ICS + LTRA [32]. As an alternative treatment low-dose ICS + LTRA was recommended in the GINA guidelines [33]. Finally, if the preschoolers were not controlled on step 3, the next step (step 4) was to consider (again without any supporting trial) adding either LABA or LTRA to a medium-dose ICS [31] or consider referral to a specialist assessment [32–34]. Only NAEPP guidelines [31] suggested additional steps for children that still with uncontrolled asthma: high-dose ICS + either LABA or LTRA for step 5; and adding OCS for step 6. The other guidelines [32–34] did not recommend the use of LABA in children under the age of 5 years. The Canadian guidelines [34] stated that until more evidence supporting their effectiveness is available, three commonly used strategies are discouraged: daily LTRA which are less effective than ICS and should remain a second-line option; stepping-up the daily dose of ICS during URTI, which remains untested in preschoolers; and the intermittent use of asthma controller mediations at the onset of symptoms (e.g., LTRA or low or medium doses of ICS) that has not been convincingly shown to reduce the number or severity of asthma exacerbations. In contrast, use of pre-emptive high-dose of ICS at the onset of symptoms is effective in reducing the severity and duration of exacerbation in preschoolers with moderate or severe viral-induced asthma; however, due to the risk for overuse and potential side effects, this treatment should be reserved for asthma specialists and only if daily ICS fails.

## Conclusions

Irrespective of the apparent phenotype, daily ICS remains the most effective strategy for preschoolers with recurrent wheezing, especially those with asthma. For infants and preschoolers with moderate or severe episodes of EVW, the use of high intermittent ICS doses significantly reduce the use of OCS. There is no evidence of effect of intermittent ICS at low-moderate doses in preschoolers with mild EVW episodes. In preschoolers with asthma, there were no significant differences between daily vs. intermittent ICS in terms of asthma exacerbations with insufficient evidence to conclude to equivalence; however, for other asthma control outcomes, daily ICS works significantly better than intermittent ICS for older children. In preschoolers with recurrent wheezing or asthma, daily low-dose ICS seem to be superior to montelukast in reducing symptoms and exacerbations and improving lung function. No RCTs of LABA or LTRA as adjunct to ICS have been published in preschoolers. Pre-emptive use of OCS by parents at home at the first sign of symptoms is not effective in preschoolers with EVW.

In terms of ICS safety, monitor linear growth is essential given that individual susceptibility to these drugs may vary considerably with attention given to reducing the dose to the lowest effective dose and selecting the molecule with least growth suppression.

### Ethics approval and consent to participate

Since it is a review paper, the study did not need ethical committee approval.

## Abbreviations

API: Asthma predictive index
ED: Emergency department
EVW: Episodic viral wheeze
FEF_25–75_: Flow expiratory forced at 25–75
FEV_1_: Forced expiratory volume in 1 s
GINA: Global Initiative for Asthma
HR: Hazard ratio
ICS: Inhaled corticosteroids
LABA: Long-acting beta2-agonits
LR: Likelihood ratio
LTRA: Leukotriene receptor antagonist
MDI: Metered-dose inhaler
MVW: Multiple-trigger wheeze
NAEPP: National Asthma Education and Prevention Program
NTT: Number need to treat
OCS: Oral corticosteroids
PEF: Peak expiratory force
RCT: Randomized clinical trial
SABA: Short-acting beta 2-agonits
